# Associations between different dimensions of prenatal distress, neonatal hippocampal connectivity, and infant memory

**DOI:** 10.1038/s41386-020-0677-0

**Published:** 2020-04-18

**Authors:** Dustin Scheinost, Marisa N. Spann, Laraine McDonough, Bradley S. Peterson, Catherine Monk

**Affiliations:** 1 Yale School of Medicine, 300 Cedar Street, New Haven, CT 06520-8043 USA; 2 Columbia University Irving Medical Center, 622 West 168th Street, New York, NY 10032 USA; 3 Brooklyn College, 2900 Bedford Avenue, Brooklyn, NY 11210 USA; 4 Institute for the Developing Mind, Children’s Hospital of Los Angeles and Keck School of Medicine, University of Southern California, 4650 Sunset Boulevard, Los Angeles, CA 90007 USA; 5 New York State Psychiatric Institute, 1051 Riverside Drive, New York, NY 10032 USA

**Keywords:** Risk factors, Hippocampus

## Abstract

Prenatal maternal distress—an umbrella concept encompassing multiple negative psychological states including stress, anxiety, and depression—is a substantial prenatal exposure. Consistent across preclinical and human studies, the hippocampus displays alterations due to prenatal distress. Nevertheless, most prenatal distress studies do not focus on multiple dimensions of, have not examined hippocampal functional connectivity in association with, and do not consider observer-based functional outcomes related to distress. We investigated the effects of different dimensions of prenatal distress in pregnant adolescents, a population at high risk for distress, in association with neonatal hippocampal connectivity and infant memory. In pregnant adolescents (*n* = 42), we collected four measures of distress (perceived stress, depression, pregnancy-specific distress, and 24-h ambulatory salivary cortisol) during the 2nd and 3rd trimesters. Resting-state imaging data were acquired in their infants at 40–44 weeks post-menstrual age. Functional connectivity was measured from hippocampal seeds. Memory abilities were obtained at 4 months using the mobile conjugate reinforcement task. Shared across different dimensions of maternal distress, increased 3rd trimester maternal distress associated with weaker hippocampal–cingulate cortex connectivity and stronger hippocampal–temporal lobe connectivity. Perceived stress inversely correlated while hippocampal–cingulate cortex connectivity positively correlated with infant memory. Increased cortisol—collected during the 2nd, but not the 3rd, trimester—associated with weaker hippocampal–cingulate cortex connectivity and stronger hippocampal–temporal lobe connectivity. Different dimensions of prenatal maternal distress likely contribute shared and unique effects to shaping infant brain and behavior.

## Introduction

A broad range of studies have documented the sequelae of exposure to different types of prenatal distress—an umbrella concept encompassing multiple negative psychological states including stress, anxiety, and depression. Studied independently, prenatal exposure to maternal stress [1, 2], anxiety [3, 4], and depression [5–7] are associated with changes in the structural and functional development of the infant brain. Similarly, maternal stress, anxiety, depression, or biological indices of distress (e.g., cortisol) are risk factors for psychiatric disorders, including attention deficit hyperactivity disorder, depression, and schizophrenia [8–11], and decrements in infant cognitive development [12]. Nevertheless, most studies published to date focus on a single dimension of prenatal distress. Thus, there is difficulty appreciating whether stress, depression, anxiety, and biological indices—such as variation in cortisol levels [2, 13]—result in the same adverse outcomes in childhood [14–16].

The hippocampus is a common candidate for investigations of the brain correlates of prenatal distress [17]. It plays an important role in response inhibition, memory, and spatial cognition [18], is altered in many psychiatric disorders [19], and contains high levels of glucocorticoid receptors [17], which regulate distress-related homeostasis. Accordingly, these receptors putatively render the hippocampus more vulnerable than most other brain areas to maternal distress that may include hypothalamic–pituitary–adrenal (HPA) axis dysregulation and atypical cortisol exposure for the fetus [20]. Consistently, the hippocampus displays alterations due to aspects of prenatal maternal distress. In rats, prenatal stress during late pregnancy alters the expression of proteins critical for neuronal plasticity in the hippocampus [21–23], and impairs spatial memory [24]. Similarly, in non-human primates, prenatal stress has been associated with 10–12% reductions in hippocampal volume [25]. Finally, in humans, increased prenatal maternal anxiety has been associated with smaller left hippocampus volumes in school-age children [26] and slower growth of both hippocampi between birth and age 6 months [3]. Despite this interest, previous studies have not examined hippocampal functional connectivity—or how the hippocampus functionally interacts with other brain regions—in neonates with respect to multiple dimensions of prenatal maternal distress. The functional interaction between anatomically distinct brain regions provides a powerful predictor of individual differences in cognition, behavior, and risk [27].

The goal of the current study is to characterize the effects of multiple dimensions of prenatal maternal distress on hippocampal functional connectivity in neonates and learning and memory in young infants. We collected four measures of distress (perceived stress, depressive symptoms, pregnancy-specific distress and anxiety, and cortisol—a biological index of stress) during both the 2nd (at 24–27 weeks of gestation) and 3rd (at 34–37 weeks of gestation) trimester in pregnant adolescents, a population at high risk for distress [28]. Using resting-state functional magnetic resonance imaging data acquired at 40–44 weeks post-menstrual age, we assessed hippocampal functional connectivity using seed connectivity. We hypothesized that 3rd trimester maternal distress would correlate inversely with functional connectivity measures from hippocampal seeds in neonates. Furthermore, we proposed that higher maternal distress and hippocampal connectivity would be associated with poorer performance on a measure of infant memory (the mobile conjugate reinforcement task), collected at 4 months [29].

## Methods

### Participants

Nulliparous pregnant adolescents, aged 14–19 years, were recruited in the 2nd trimester (13–28 weeks) through the Departments of Obstetrics and Gynecology at Columbia University Irving Medical Center (CUIMC), Weill Cornell Medical College, and flyers posted in the CUIMC vicinity as part of a longitudinal study examining adolescent pregnancy behaviors and infant outcomes. The pregnant adolescents received routine prenatal care and had no major health problems at the time of recruitment. Participating mothers provided informed consent and the study procedures were approved by the Institutional Review Boards of the New York State Psychiatric Institute and of CUMC. If they were under 18 years of age, they completed an assent form and their parent signed a consent form. Participants were excluded if they acknowledged the use of recreational drugs, tobacco, alcohol, or medications with an effect on cardiovascular function, or lacked fluency in English. Of the 72 infants who underwent MRI scanning, 46 had usable functional MRI data (see the Motion Analysis section in Supplemental Material); 31 of those infants had all measures of maternal distress during the 2nd trimester, and 42 of those infants had all measures of maternal distress during the 3rd trimester. All infants had two runs of resting-state data (3 min 44.4 s each; voxel size 3.4 × 3.4 × 5 mm^3^) for analysis.

### Distress measures

In the 2nd (at 24–27 weeks of gestation) and 3rd (at 34–37 weeks of gestation) trimesters, we collected four measures of maternal distress: the Perceived Stress Scale (PSS), the Reynolds Adolescent Depression Scale (RADS), the Pregnancy Distress Questionnaire (PDQ), and 24-h salivary cortisol (AUC for 5 timepoint diurnal collection). The PSS was also administered postnatally when the infant was 4 months old. See Supplementary Material for more details.

### The mobile conjugate reinforcement task

The mobile conjugate reinforcement task was administered to 4-month-old infants in a laboratory setting as previously described [29]. This task is well-established as a reliable and valid measure of learning and memory in young infants [30]. From this task, we used the long-term retention ratio, which is calculated by dividing infant kick rate during the long-term retention period on Day 2 by infant kick rate during the baseline period on Day 1. See Supplementary Material for more details.

### Common space registration

A study-specific common space template was created using the anatomical images from all study participants. To create this template, first, anatomical images were skull stripped using FSL (https://fsl.fmrib.ox.ac.uk/fsl/) and any remaining non-brain tissue was manually removed. Next, using BioImage Suite [31], anatomical images were linearly aligned to a single infant anatomical scan from an independent study [32] using a 12 parameter affine registration by maximizing the normalized mutual information between images. Finally, anatomical images were non-linearly registered to an evolving group average template in an iterative fashion using a previously validated algorithm [33]. After the anatomical scans were registered to the common space template, functional images were rigidly aligned to the anatomical images. All transformation pairs were calculated independently and combined into a single transform, warping the single participant results into common space. This single transformation allows the individual participant images to be transformed to common space with only one transformation, thereby reducing interpolation error.

### Connectivity processing

Motion correction was performed using SPM8 (http://www.fil.ion.ucl.ac.uk/spm/). Images were warped into 3 mm^3^ common space using the non-linear transformation described above and cubic interpolation. Next, images were iteratively smoothed until the smoothness of any image had a full-width half-maximum of approximately 8 mm using AFNI’s 3dBlurToFWHM (http://afni.nimh.nih.gov/afni/). This iterative smoothing reduces motion-related confounds [34]. Several covariates of no interest were regressed from the data, including linear and quadratic drifts, mean cerebral-spinal-fluid (CSF) signal, mean white matter signal, and mean gray matter signal. For additional control of possible motion-related confounds, a 24-parameter motion model (including six rigid-body motion parameters, six temporal derivatives, and these terms squared) was regressed from the data. The functional data were temporally smoothed with a Gaussian filter (approximate cutoff frequency = 0.12 Hz). A dilated gray matter mask was applied to the data so only voxels within gray matter were used in further calculations. The CSF, white matter, and gray matter masks were manually defined on the reference brain.

### Seed connectivity

We assessed whole brain seed connectivity, independently, from the right and left hippocampus, shown in Fig. S1A. Seeds were manually defined on the reference brain and included the hippocampal head and body. The time course of the reference region in a given participant was then computed as the average time course across all voxels in the seed region. This time course was correlated with the time course for every other voxel in gray matter to create a map of *r*-values, reflecting seed-to-whole-brain connectivity. These *r*-values were transformed to *z*-values using Fisher’s transform yielding one map for each seed and representing the strength of correlation with the seed for each participant.

### Experimental design and statistical analyses

Our primary analyses are those associating measures of maternal distress collected during the 3rd trimester with connectivity of the hippocampi to the whole brain. We consider these analyses primary for two reasons. First, as functional networks rapidly develop over the 3rd trimester making those circuits highly vulnerable to prenatal exposures [35–37], we hypothesized that 3rd trimester distress would have the greatest impact on hippocampal functional connectivity. Second, we have a greater sample size of those with distress measures collected in the 3rd trimester compared to those with distress measures collected in the 2nd trimester. Analyses of measures of distress collected during the 2nd trimester are presented as exploratory. See SI for sample size breakdown for each measure.

Demographic and behavioral data were analyzed using either standard Chi-squared test statistics or Fisher’s exact test for categorical data. Continuous data were analyzed using either *t*-tests or Mann–Whitney *u*-tests and Spearman rank correlation when a normal distribution could not be assumed to compare groups. For infants missing the long‐term retention ratio, mean imputation was used. For analyses, using imputed values, an extra regressor denoting which impute values was included. All analyses were performed using SPSS 23 (IBM, New York); *p*-values <0.05 were considered statistically significant.

Imaging data were analyzed using voxel-wise linear models controlling for sex, postmenstrual age, and scanner upgrade with all three covariates included in a single model. Imaging results are thresholded at *p* < 0.05, with all maps corrected for multiple statistical comparisons across gray matter using cluster-level correction estimated via AFNI’s 3dClustSim (version 16.3.05) with 10,000 iterations, an initial cluster forming threshold of *p* = 0.001, the gray matter mask applied in preprocessing, and a mixed-model spatial autocorrelation function (ACF). Parameters for the spatial ACF were estimated from the residuals of the voxel-wise linear models using 3dFWHMx.

## Results

### Demographic characteristics

The maternal and neonatal demographic characteristics are summarized in Tables S1 and S2. The average age of the pregnant women was 17 years. The majority was Hispanic (86%) and a over half completed high school (52%). The majority of infants were male (68%) and delivered vaginally (77%). All infants were appropriate size for gestational age (birth weight: M = 3161.2, SD = 430.6 g, gestational age at birth: M = 39.3, SD = 1.4 weeks) and were scanned at an average 42.4 (SD = 1.7) weeks postmenstrual age (PMA). There were no differences in demographic or distress measures between neonates with and without usable functional data (*p* > 0.15, for all). Average distress measure scores for the participants are reported in Table S3. Correlations between the individual distress measures are presented in Table S4.

### Associations of maternal distress in the 3rd trimester with hippocampal connectivity in neonates

#### Associations of maternal perceived stress with hippocampal connectivity in neonates (n = *42*)

**Fig. 1:**
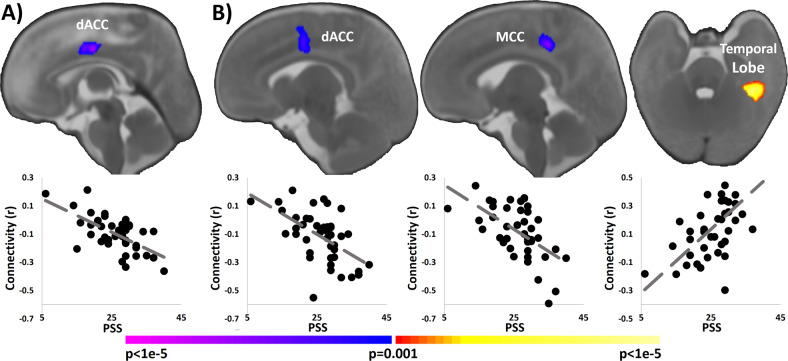
Association of prenatal perceived stress and hippocampal connectivity. **a** Higher levels of perceived stress scale (PSS) in the 3rd trimester were associated with weaker infant connectivity between the left hippocampus and the dorsal anterior cingulate cortex (dACC). **b** Higher PSS associated with weaker connectivity between the right hippocampus and dACC, with weaker connectivity between the right hippocampus and middle cingulate cortex (MCC), and with stronger connectivity between the right hippocampus and the temporal lobe. Scatterplots below the images visualize the distribution of the observed data points for average infant connectivity in the detected regions plotted against maternal perceived stress during the 3rd trimester.

Higher maternal perceived stress as measured by the PSS was associated with weaker connectivity between the left hippocampus and dorsal anterior cingulate cortex (dACC; Fig. 1a). Similarly, higher perceived stress associated with weaker connectivity between the right hippocampus and dACC, weaker connectivity between the right hippocampus and middle cingulate cortex (MCC), and stronger connectivity between the right hippocampus and the left temporal lobe (Fig. 1b).

As the correlation between maternal perceived stress during the 3rd trimester and at 4 months showed a weak to moderate effect size (*r* = −0.34, *p* = 0.06, df = 30, *n* = 32), we investigated the specificity of our finding to prenatal stress, rather than postnatal stress. As the PSS only includes stress in the previous month, the 4-months assessment is sufficiently far away from the prenatal period that the measure of stress only reflects stress during the post-partum period (within 1 month of the time of assessment) and not stress during the prenatal period. Using the average connectivity values extracted from regions of interest based on the initial analyses, we performed *post-hoc* partial correlation, controlling for postnatal PSS collected. All associations exhibited a similar effect size with the absolute values of the correlation coefficients ranging from 0.47 to 0.67 (*p*-value range = 0.007–0.000002; df = 29, *n* = 32). Additionally, we did not observe any associations between postnatal maternal stress and hippocampal connectivity using voxel-wise correlations.

#### Associations of maternal depression with hippocampal connectivity (n = *42*)

**Fig. 2:**
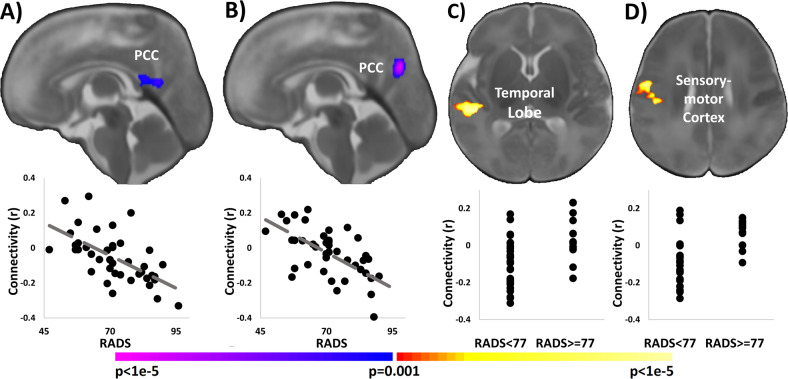
Association of maternal depression and hippocampal connectivity. Higher depressive symptoms in the 3rd trimester as measured by the Reynolds Adolescent Depression Scale (RADS) were associated with weaker infant connectivity between the **a** left and **b** right hippocampus and the posterior cingulate cortex (PCC). When dichotomized based on clinical cutoffs, neonates from mothers with prenatal depression exhibited greater connectivity **c** between the left hippocampus and the right temporal lobe and **d** between the right hippocampus and the right sensory-motor cortex. Scatterplots below the images visualize the distribution of the observed data points for average infant connectivity in the detected regions plotted against maternal depressive symptoms or clinical group.

Higher maternal depressive symptoms as measured by the RADS associated with weaker connectivity between the left hippocampus and posterior cingulate cortex (PCC; Fig. 2a) and with weaker connectivity between the right hippocampus and PCC (Fig. 2b). When comparing dichotomized groups based on the RADS clinical cutoff scores, neonates from mothers with prenatal depression exhibited greater connectivity between the left hippocampus and the right temporal lobe (Fig. 2c) and greater connectivity between the right hippocampus and the right sensory-motor cortex (Fig. 2d).

#### Associations of maternal pregnancy-specific distress with hippocampal connectivity (n = *42*)

**Fig. 3:**
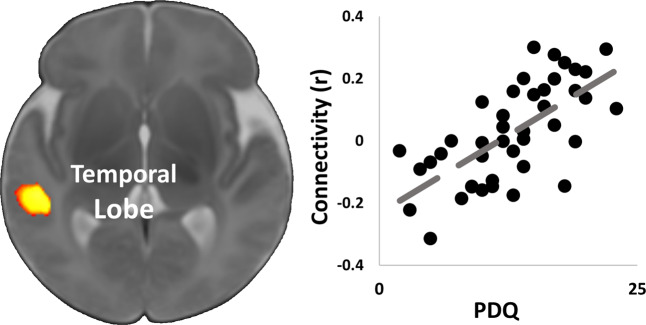
Associations of pregnancy-specific distress and hippocampal connectivity. Higher maternal pregnancy-specific distress as measured by the Pregnancy Distress Questionnaire (PDQ) associated with stronger connectivity between the left hippocampus and right temporal lobe. Scatterplots next to the images visualize the distribution of the observed data points for average infant connectivity in the detected regions plotted against pregnancy-specific distress during the 3rd trimester.

Higher maternal pregnancy-specific distress as measured by the PDQ associated with stronger connectivity between the left hippocampus and right temporal lobe (Fig. 3).

#### Associations of maternal prenatal cortisol with hippocampal connectivity in neonates (n = *25*)

We did not observe any associations between maternal cortisol collected during the 3rd trimester and left or right hippocampal connectivity.

#### Controlling for co-occurring dimensions of maternal distress

Using the average connectivity values extracted from regions of interest based on the initial analyses, we performed *post-hoc* partial correlation associating maternal distress and hippocampal connectivity, independently controlling for the other dimensions maternal distress measures. For example, based on average connectivity from the regions in Fig. 1, we calculated the associations between maternal perceived stress with hippocampal connectivity, controlling for maternal depression, pregnancy-specific distress, and cortisol. All maternal distress–hippocampal connectivity associations had similar effect sizes when controlling for co-occurring maternal distress with the absolute values of the correlation coefficients ranging from 0.48 to 0.67 (*p*-value range = 0.002–0.000007; df = 36, *n* = 42).

#### Controlling for 2nd trimester maternal distress

Additionally, we performed *post-hoc* partial correlation associating maternal distress and hippocampal connectivity, independently controlling for the same distress measure collected during the 2nd trimester. For example, we calculated the associations between 3rd trimester, maternal perceived stress with hippocampal connectivity, controlling 2nd trimester, maternal perceived stress. Associations with perceived stress and pregnancy-specific distress had similar effect sizes with the absolute values of the correlation coefficients ranging from 0.43 to 0.65 (*p*-value range = 0.007–0.000007; df = 36, *n* = 39). Associations with maternal depression were no longer significant with correlation coefficients ranging from 0.4 to 0.45 (*p*-value range = 0.26–0.33; df = 6, *n* = 9). We attribute this lack of significance to the low numbers of participants with depression measures for both the 2nd and 3rd trimesters.

### Associations of neonatal hippocampal connectivity and infant memory

Given the hippocampus’s critical involvement in long-term memory, we correlated 3rd trimester distress and neonatal hippocampal connectivity with a measure of infant memory and learning, the long-term retention ratio from the mobile conjugate reinforcement task, collected at 4 months. Higher maternal perceived stress during the 3rd trimester associated with lower long-term retention ratio on the mobile conjugate reinforcement task administered at 4 months (*ρ* = −0.34, *p* = 0.04, df = 34, *n* = 36, Fig. S2). Neither the prenatal depressive symptoms nor pregnancy-specific distress nor cortisol associated with the long-term retention ratio. Next, given the association between perceived stress and infant memory, we correlated the long-term retention ratio with the average connectivity values extracted from regions of interest shown in Fig. 1. Connectivity between the right hippocampus and the dACC (*ρ* = 0.33, *p* = 0.05, df = 34, *n* = 36, Fig. S2) associated with infant memory, such that higher connectivity resulted in higher long-term retention. We did not observe associations with the right hippocampus to bilateral temporal lobe or to the MCC findings or with the left hippocampus. Finally, we tested the mediating effects of hippocampal connectivity on the association between prenatal perceived stress and infant memory (*t* = −1.84, *p* = 0.075, df = 35, *n* = 36, Cohen’s *d* = 0.63).

### Exploratory analyses

#### Maternal distress measures collected during the 2nd trimester

**Fig. 4:**
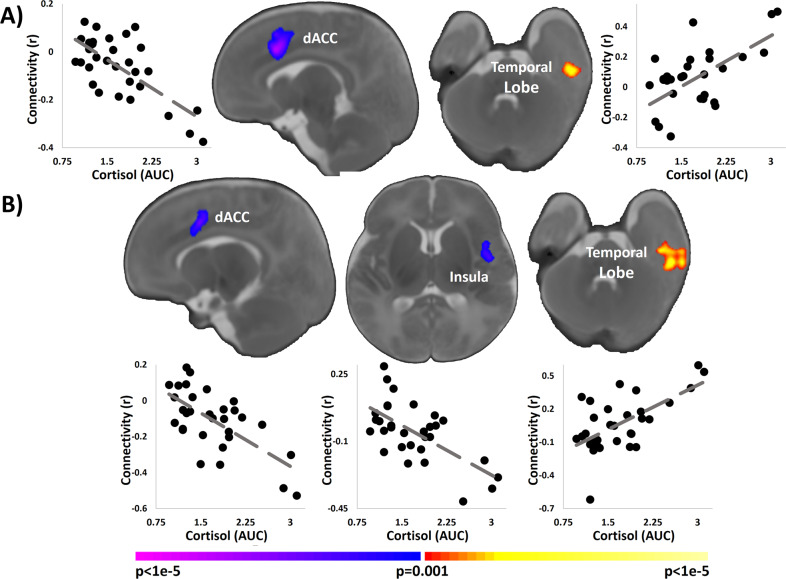
Association of 2nd trimester cortisol and hippocampal connectivity. **a** Higher maternal cortisol associated with weaker connectivity between the left hippocampus and the dorsal anterior cingulate cortex (dACC) and with stronger connectivity between the left hippocampus and left temporal lobe. **b** Higher maternal cortisol associated with weaker connectivity between the right hippocampus and the dACC, with weaker connectivity between the right hippocampus and the insula, and with stronger connectivity between the right hippocampus and left temporal lobe. Scatterplots next to the images visualize the distribution of the observed data points for average infant connectivity in the detect regions plotted against maternal cortisol during the 2nd trimester.

In an exploratory analysis, we repeated the analyses using distress measures collected in the 3rd trimester with those collected during the 2nd. We did not observe any associations between the PSS (*n* = 41), RADS (*n* = 9), or PDQ (*n* = 41) and the left or right hippocampal connectivity. Higher maternal cortisol associated with weaker connectivity between the left hippocampus and the dACC and with stronger connectivity between the left hippocampus and left temporal lobe (Fig. 4a; *n* = 31). Similar associations were observed for the right hippocampus (Fig. 4b; *n* = 31). Additionally, higher maternal cortisol associated with weaker connectivity between the right hippocampus and left insula (Fig. 4b, *n* = 31).

#### Spatial overlap of effects

We computed the overlap of all detected maternal distress–hippocampal connectivity associations. With the exception of a small cluster of overlap between the maternal depression and pregnancy-specific distress clusters in the right temporal lobe (96 voxel; 53 mm^3^), all clusters were spatially distinct (Fig. S3).

## Discussion

In this prospective study, we investigated the effects of different dimensions of prenatal maternal distress on hippocampal functional connectivity in neonates and infant memory at 4 months. We measured perceived stress, depression, pregnancy-specific distress, and cortisol during the 2nd and 3rd trimesters, functional connectivity in the hippocampal network in neonates at 40–44 weeks PMA, and infant memory at 4 months. Associations between 3rd trimester maternal distress and hippocampal connectivity in the cingulate cortex (e.g., inverse correlations) and temporal lobes (e.g., positive correlations) were observed across different dimensions of maternal distress (i.e., perceived stress, depression, and pregnancy-specific distress). Yet, each dimension mapped onto unique spatial locations in each region and effect size were similar when controlling for co-occurring distress. Perceived stress correlated inversely and hippocampal connectivity correlated positively with infant memory. Maternal cortisol collected during the 2nd, but not the 3rd, trimester correlated with the strength of hippocampal connectivity in the ACC, insula, and temporal lobe. These results add to the growing literature of the impact of prenatal maternal distress on the developing connectome [36] suggesting that different dimensions of distress may have both shared and unique effects [38].

### Effects of maternal prenatal distress on neonatal connectivity

Measures of distress related to stress (i.e., perceived stress and cortisol) mapped onto hippocampal–ACC connectivity. The hippocampal–ACC network has been well-studied in children and adults. It plays vital roles in memory and emotional state processing [39, 40] and is commonly altered in neuropsychiatric disorders of chronic stress [41]. The hippocampus connects to the ACC through polysynaptic connections involving the mammillary bodies/hypothalamus and thalamus [17, 39]. Acute stress modulates brain activity in these areas, leading to region-specific alterations and cognitive deficits (i.e., memory) that can be worsened over time with chronic stress [42]. Overall, our results are consistent with previous research investigating alterations in the hippocampal–ACC network. Critically, they extend our knowledge of the hippocampal–cingulate network by highlighting similarities between the role of stress and memory in the developing brain and the role of stress and memory in older children and adults.

We observed associations between weaker hippocampal–PCC connectivity and prenatal distress only when using depressive symptoms. The hippocampus and PCC are both part of the default mode network [43], which is commonly altered in depression [44, 45]. Although speculative, these observations may suggest that alterations in hippocampal–PCC connectivity may be more specific to depression than other dimensions of distress. Longitudinal studies in which maternal depressive symptoms (collected prenatally) are associated with the offspring hippocampal–PCC connectivity would be needed to further test this hypothesis.

All measures of maternal distress (perceived stress, depression, pregnancy-related distress, and cortisol) correlated positively with the strength of connectivity between the hippocampus and the temporal lobe, suggesting that the alterations in temporal lobe connectivity is shared across the different dimensions of distress. Nevertheless, the associations do not have the same anatomical locations, with the exception of a small region associated with both pregnancy-specific distress and depression. The temporal lobe is involved in a wide range of cognitive processes [46]. As different dimensions of prenatal distress have been associated with distinct cognitive process, these results suggest that effects of prenatal distress on the developing brain could occur within different sub-pathways of the temporal lobe, with the mediating biological pathways and associated genes of risk to be determined.

### A role for the hypothalamic–pituitary–adrenal axis

The maternal hypothalamic–pituitary–adrenal (HPA) axis—the central stress response system [47]—is a primary biological mechanism by which maternal distress affects the fetus [38]. Distress activates the HPA axis, leading to a change in glucocorticoid receptors in the hippocampus via cortisol and other glucocorticoid signaling pathways [48]. As cortisol can cross the placental barrier, similar glucocorticoid signaling pathways affect fetal development [49]. For example, placental studies of mRNA expression show that prenatal distress can alter the expression of genes for glucocorticoid signaling [50, 51]. Taken together, prenatal maternal distress may lead to alterations in neonatal hippocampal connectivity through the HPA axis and glucocorticoid signaling, which can be assessed in future studies. This would explain our findings of the association between 2nd trimester maternal cortisol and the strength of hippocampal connectivity in the ACC, insula, and temporal lobe.

In the absence of maternal distress, HPA axis function is highly affected by pregnancy and plays a critical role in the normal development of the fetus. Pituitary gland size increases drastically throughout the 3rd trimester, peaking at 120% of its normal volume during parturition [52, 53], and corticotropin-releasing hormone (CRH, which stimulates cortisol release through adrenocorticotropic hormones) increases exponentially during the 3rd trimester, starting approximately at 32 weeks gestation [54]. The placenta also functions as an endocrine regulator, producing CRH by stimulating greater production of the cascade of steroid hormones (e.g., adrenocorticotropic hormone and cortisol) to the developing fetus [12, 55] In the placenta, cortisol creates a positive feedback loop by further stimulating CRH production over the course of gestation [12, 55]. Given HPA changes in maternal-fetal regulation during pregnancy, it is plausible that HPA activity may not reflect distress to the same extent as its activity in early trimesters and in non-pregnant individuals. Therefore, that the 3rd trimester cortisol was collected during this period of rapid parturition-related changes in the HPA axis (i.e., 34–37 weeks gestation) may explain the lack of associations between 3rd trimester cortisol and both hippocampal functional connectivity and self-reported measures of distress.

### Strengths and limitations

Our study has several key strengths. We acquired data prospectively beginning in the 2nd trimester of pregnancy and continued collecting data in infants through 4 months. By controlling for postnatal maternal distress and acquiring imaging data in the infants soon after birth, we were able to attribute functional characteristics largely to prenatal—rather than postnatal—factors [2, 56, 57]. Finally, we included multiple dimensions of distress collected in both the 2nd and 3rd trimester, which allowed us to investigate the shared and unique effects of multiple dimensions of prenatal distress on hippocampal functional connectivity. While our results suggest that similar, but different, forms of prenatal exposures can lead to similar brain outcomes (in that similar large-scale regions were associate with dimensions of distress), it remains important to not broadly pool exposures as brain outcomes may vary across even similar exposures.

Our study has several limitations warranting discussion. Our maternal sample consisted of adolescents, who are at higher risk for prenatal distress [28]. Thus, the observed associations may not generalize to other pregnant populations. Similarly, a majority of the sample is male. Sex differences in the effect of prenatal distress on brain connectivity, and hippocampal development have previously been reported [36, 58]. Investigations into the role of sex in the current study necessitate a larger sample size for greater statistical power. Likewise, our sample size likely limited the statistical power of the mediation analysis. Despite the longitudinal nature of our study, we only had a single imaging and behavioral assessment time point. Developmental trajectories of functional connectivity may have provided a better assessment of the influences of maternal distress on the developing brain [59]. Finally, as we did not have a measure of anxiety in our sample, we could not investigate an important dimension of distress. While the PDQ does include items relevant to anxiety, further work is needed to elucidate potential effects due to maternal anxiety.

### Conclusions

Consistent across multiple dimensions of maternal distress collected during pregnancy, we showed that 3rd trimester distress likely influences the developing brain. Our findings provide evidence that prenatal distress is associated with hippocampal networks implicated in stress, long-term memory formation, and psychiatric illness. Together, these results suggest that different dimensions of maternal distress have both shared and unique effects on the hippocampal connectivity with the cingulate cortex and the temporal lobes. Future studies should continue to clarify the shared and unique ways that different dimensions of distress map onto the developing brain, possibly using outcome measures collected longitudinally and predictive modeling methods.

## Funding and disclosure

This work was supported by the National Institute of Mental Health (MH093677-05), the National Center for Advancing Translational Sciences (KL2 TR001874 and 000081), and the Marilyn and James Simons (MJS) Foundation (Whitaker Scholar Developmental Neuropsychiatry Program). The authors declare no competing financial interests.

## Supplementary information

SI
