# Supplementary material for: Associations between different dimensions of prenatal distress, neonatal hippocampal connectivity, and infant memory
Source: Neuropsychopharmacology. 2020 Apr 18;45(8):1272–9. doi: 10.1038/s41386-020-0677-0 (PMC7297970; doi:10.1038/s41386-020-0677-0)
Supplement: Supplementary file 1 — SI [file 41386_2020_677_MOESM1_ESM.docx]

**Methods**

**Participants**

The imaging component of the study was embedded in a larger study involving data collection at multiple time points during pregnancy and the post-natal period. As part of the larger study, 72 infants underwent MRI leading to 46 infants with both usable anatomical and functional data (7 did not have usable anatomical data and 19 did not have usable functional data). From these 46 infants, 42 had PSS, RADS, and PDQ at the 3rd trimester; 25 had cortisol at the 3rd trimester, 41 had PSS and PDQ at the 2nd trimester; 9 had RADS at the 2nd trimester, 31 had cortisol at the 3rd trimester; 32 had the PSS at 4-months; and 36 had the mobile conjugate reinforcement task. Thus for our primary goal of investigating the shared and unique components of prenatal distress only the 3rd trimester data had a reasonable sample size for all measures.

**Distress measures**

The PSS is a well-established 14-item self-report assessing the degree to which situations are appraised as threatening or demanding. The PSS is a widely used and highly reliable perceived stress scale with culturally adjusted norms and has also been used to determine prenatal stress (Shelton et al., 2015; Solivan et al., 2015; Rieger and Heaman, 2016). Higher scores are indicative of a higher level of perceived stress.

The RADS is a 30-item self-report questionnaire assessing depressive symptoms in adolescents, aged 13 to 18 years (Reynolds and Mazza, 1998). Each item requires the subject to report the frequency of occurrence of a particular affective state or experience. Higher scores are indicative of higher endorsement of depressive symptoms. A cutoff score of 77 or above indicates a clinically relevant level of depression that impairs the individual’s daily functioning.

The PDQ is a reliable and valid measure of pregnancy-specific stress and anxiety (maternal fears and worries related to pregnancy) (Yali and Lobel, 1999). The PDQ is a 12-item scale that provides three subscales: concerns about birth and the baby, concerns about weight/body image, and concerns about emotions and relationships. Higher Scores on the PDQ are indicative of a higher level of pregnancy-specific stress and anxiety.

Cortisol is a steroid hormone that mediates many aspects of the stress response in adults and has been shown to negatively impact neonatal development (de Weerth et al., 2003; Davis et al., 2007). Forty-eight hour, salivary cortisol collection was timed to begin during the 1st day of each study session. Subsequent samples on the 2^nd^ day of collection were as follows: at waking; 45 minutes, 2.5 hours, 3.5 hours, and 8 hours after waking; and at 10 PM or before going to bed. Cotton used for each sample was kept in a bottle with a Medication Event Monitoring System (MEMS) track cap (Aardex, Union City, CA), which records the time of opening and has been shown to help adolescents and adults comply with sampling protocols (Adam and Kumari, 2009). Once used, the cotton was placed in a Salivette tube (Sarstedt, Newton, NC). After return to the lab, samples were kept frozen at −80°C until assayed using a commercial ELISA/ EIA kit optimized for saliva (Salimetrics, State College, PA). All salivary cortisol values were used to calculate the area under the curve (a measure of the dynamics of cortisol) and this measure was use for analysis.

**The mobile conjugate reinforcement task**

In this task administered over a two day period, one end of a ribbon is tied around an infant’s ankle and the other end is connected to a mobile hanging over their crib. Through experience with this set-up, the infant learns the contingency between kicking and movement of the mobile. After a delay, the task is repeated, and retention is measured by examining whether the infant kicks more during the retention phase than at baseline. Both the speed of learning and length of retention increase with age.

Briefly, each session began with a 3-minute non-reinforcement phase (baseline), followed by a 9-minute reinforcement phase (three 3-minute learning blocks), and a final 3-minute non-reinforcement phase (immediate retention). During periods of non-reinforcement, the other end of the ribbon that was tied to the infant’s ankle was attached to the metal bracket without the mobile hanging from it. In this arrangement, the mobile remained in full view, but any movement of the leg with the ribbon on it had no effect on the mobile. During periods of reinforcement, the mobile was moved to the same bar as the ribbon. Thus, kicking the leg with the ribbon attached to it caused the mobile to bounce. On Day 1, the initial 3-minute period of non-reinforcement (baseline) provides a measure of the infant’s baseline kick rate, and the final 3-minute period of non-reinforcement provides a measure of the infant’s immediate retention. On Day 2, kicking during the initial period of non-reinforcement (Day 2 baseline) reflects the infant’s long-term (24-hr) retention of the contingency. Trained coders used the videotapes to count the number of times per minute that an infant kicked the leg with the ribbon attached to it. A kick was defined as a linear or circular movement of the foot and leg retraced in a continuous motion back to the point of origin (Rovee and Rovee, 1969). Previously reported inter-rater reliability for coders using this sample was high (α=0.95) (Merz et al., 2017). From this task, we used the long-term retention ratio, which is calculated by dividing infant kick rate during the long‐term retention period on Day 2 by infant kick rate during the baseline on Day 1.

**Imaging procedures**

Infants were scanned within the first weeks of postnatal life relative to conception (mean=42.4; SD=1.6 weeks gestational age). They were fed, swaddled, and acclimated to the scanning environment and scanner noise by listening to a tape recording of the scanner sounds played before each pulse sequence. The infants were given time to fall asleep, without the use of sedatives, while lying on the scanner bed before the start of each sequence. Foam and wax ear plugs along with ear shields (Natus Medical Inc., San Carlos, CA) were applied to dampen scanner noise. MRI-compatible EKG leads were placed on the infant’s chest, and a pulse oximetry sensor was placed on the infant’s toe. Heart rate and oxygen saturation were continually monitored during the scan (InVivo Research, Orlando, FL).

**Imaging Parameters**

Images were collected using a 3 Tesla General Electric (GE) Signa MRI scanner (Milwaukee, Wisconsin) and an 8-channel head coil. High resolution anatomical T2-weighted images were acquired using a 2D, multiple-shot, fast spin echo pulse sequence that employed PROPELLER (Periodically Rotated Overlapping Parallel Lines with Enhanced Reconstruction) to reduce motion artifacts in reconstructed MR images (Pipe, 1999): repetition time (TR)=10,000 ms; echo time (TE)=130 ms; echo train length (ETL)=32; matrix size=192×192; field of view (FOV)=190×190 mm; phase FOV=100%; slice thickness=1.0mm; number of excitations (NEX)=2. The spatial resolution of the T2-weighted images was 1mm^3^. Functional images were acquired using a standard echo-planar imaging sequence: TR=2,200 ms; TE=30 ms; matrix size=64×64; FOV=190×190 mm; phase FOV=100%; slice thickness=5.0mm, contiguous; number of slices=24; bandwidth=7812.5 Hz.

**Motion Analysis**

As motion and amount of data for analysis affects functional connectivity measures (Van Dijk et al., 2012; Noble et al., 2017), we employed a strict inclusion criterion and required that participants had 2 runs with an average frame-to-frame motion of less than 0.1 mm as previously reported (Spann et al., 2018). We detected no significant correlations between head motion and maternal distress (PSS: *r*=0.07, p=0.67, df=38; PDQ: *r*=-0.02, p=0.9, df=38; RADS: *r*=0.15, p=0.36, df=38).

**Results**

Birth weight was associated with 2^nd^ trimester PDQ (*r*=-0.44, *p*=0.004, df=38). Birth length was associated with 3^rd^ trimester PSS (*r*=-0.392, *p*=0.02, df=38). Delivery type and complications were associated with 3^rd^ trimester cortisol (F_df=2,22_=4.7, *p*=0.02; F_df=1,21_=10.9, *p*=0.003, respectively). No other significant correlations were found between the distress measures and the demographic variables. The PDQ in the 2^nd^ and 3^rd^ trimester had a high correlation (see Table 4). The other distress measures (e.g., PSS, RADS, and cortison) also independently had high correlations with their 2^nd^ and 3^rd^ trimester scores. The PDQ in both trimesters moderately correlated with the PSS and RADS in either or both trimesters.

**Hippocampal connectivity in neonates**

Activity in the left hippocampus significantly correlated with local activity in the amygdala, basal ganglia, thalamus, and insula (Figure S1B). Significant negative connectivity (i.e. inverse correlation) with the left hippocampus was observed for bilateral sensorimotor cortex and visual cortex. Connectivity patterns for the right hippocampus were similar to the connectivity patterns for the left hippocampus (Figure S1C).


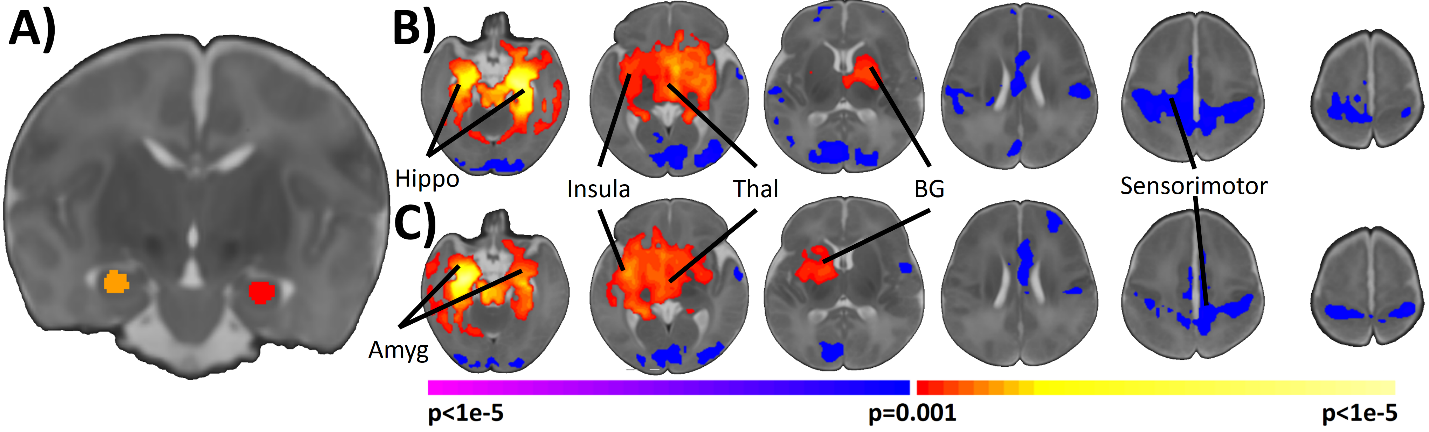


**Figure S1: Hippocampal connectivity in neonates. A) Regions of interest used for seed connectivity.** The right and left hippocampal seeds are shown in orange and red, respectively. **B) Left and C) right hippocampal connectivity.** The hippocampus (Hippo) is functionally connected primarily to bilateral amygdala (Amyg), bilateral insula, ipsilateral basal ganglia (BG), the bilateral thalamus (Thal), and the sensorimotor regions.


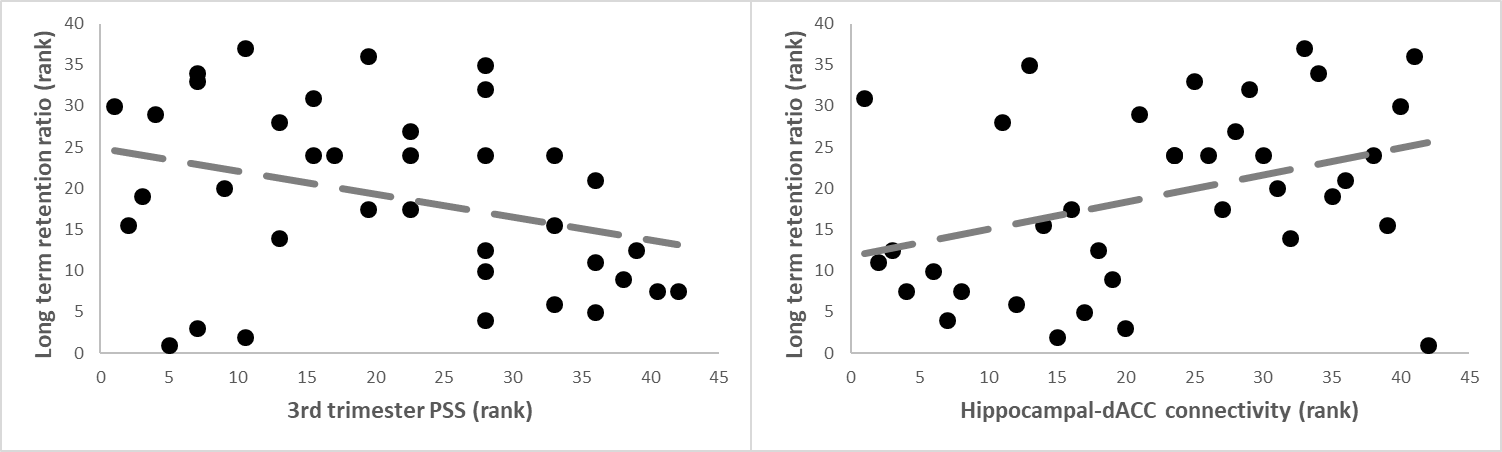


**Figure S2: Associations between infant memory and both (left) 3^rd^ trimester PSS and (right) hippocampal-dACC connectivity.**


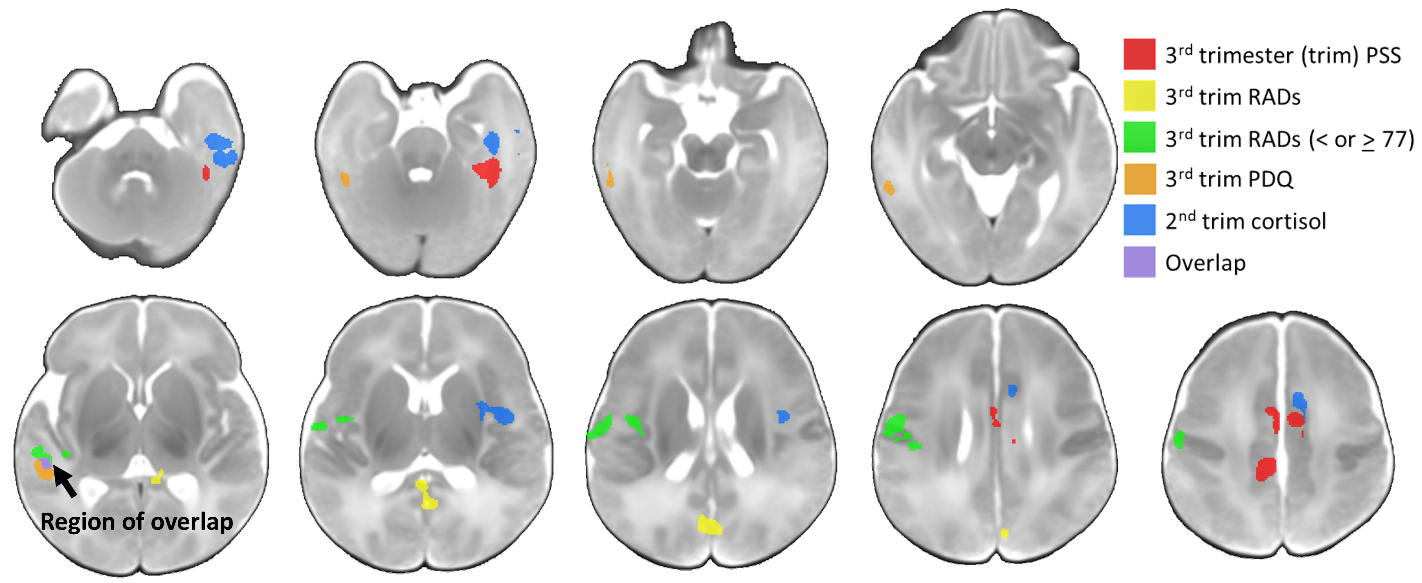


**Figure S3: Spatial overlap of significant clusters.** To highlight the unique spatial locations of how each different dimensions of distress maps onto the infant brain, all significant maternal distress-hippocampal connectivity association (from Figures 1, 2, 3, and 4) were overlaid onto a single template. With the exception of a small cluster of overlap between the maternal depression and pregnancy-specific distress clusters in the right temporal lobe (see arrow; 96 voxel; 53 mm^3^), all clusters were spatially distinct.

| Table S1. Maternal Demographics | |
| --- | --- |
| **Variables** | **n (%)** |
| Age at Delivery, years^a^ | 17.7 + 3.2 |
| Years of Education |  |
| 9^th^ grade | 5 (11.4) |
| 10^th^ grade | 7 (15.9) |
| 11^th^ grade | 9 (20.5) |
| 12^th^ grade or higher | 23 (52.3) |
| Race |  |
| Not Hispanic/Latina | 6 (13.6) |
| Hispanic/Latina | 38 (86.4) |
| Type of Delivery |  |
| Vaginal Spontaneous | 18 (45.0) |
| Assisted Vaginal^b^ | 13 (32.5) |
| Emergent Cesarean Section^c^ | 9 (22.5) |
| Pregnancy Complications^d^ |  |
| None | 32 (82.1) |
| Complications | 7 (17.9) |
| All mothers of Hispanic ethnicity were coded together.  ^a^Data presented as mean + SD.  ^b^Assisted vaginal includes induction and augmentation.  ^c^Emergent cesarean sections were due to arrest of dilatation/descent (n=6), breech presentation (n=1), and unknown reason (n=1).  ^d^Complications occurred across delivery types: vaginal spontaneous (n=1) due to chorioamnionitis, assisted vaginal (n=1) due to group B streptococcus, and emergent cesarean section (n=2) due to acute nephritic syndrome and chorioamnionitis. | |
|  | |

| Table S2. Neonatal Demographics | |
| --- | --- |
| **Variables** | **Mean** + **SD** |
| Gestational Age at Birth, weeks | 39.3 + 1.3 |
| Birth Weight, g | 3161.2 + 430.6 |
| Birth Head Circumference, cm | 33.9 + 1.4 |
| Birth Length, cm | 50.9 + 2.1 |
| Apgar 1 minute | 8.6+ 0.8 |
| Apgar 5 minute | 9.0 + 0.2 |
| Postmenstrual Age at Scan, weeks | 42.4 + 1.7 |
| Gender^a^ |  |
| Male | 30 (68.2) |
| Female | 14 (31.8) |
| Note: ^a^Data presented as n (%). | |

| Table S3. Average Prenatal Stress Values in Both Trimesters | |
| --- | --- |
| **Variables** | **Mean** + **SD** |
| 2^nd^ Trimester PSS | 27.8 + 7.5 |
| 2^nd^ Trimester RADS | 68.4 + 11.1 |
| 2^nd^ Trimester RADS >= 77^a^ | 2 (17) |
| 2^nd^ Trimester PDQ | 8.5 + 4.0 |
| 2^nd^ Trimester Cortisol | 1.7 + 0.6 |
|  |  |
| 3^rd^ Trimester PSS | 26.1 + 6.8 |
| 3^rd^ Trimester RADS | 71.7 + 11.6 |
| 3^rd^ Trimester RADS >= 77^a^ | 15 (36) |
| 3^rd^ Trimester PDQ | 12.9 + 5.3 |
| 3^rd^ Trimester Cortisol | 2.2 + 0.8 |
| Note: ^a^Data presented as n (%). | |

| Table S4. Correlations between dimensions of maternal distress^a^ | | | | | | | | |
| --- | --- | --- | --- | --- | --- | --- | --- | --- |
| **Variables** | **1** | **2** | **3** | **4** | **5** | **6** | **7** | **8** |
| **1** PDQ – 2^nd^ Trimester | -- |  |  |  |  |  |  |  |
| **2** PDQ – 3^rd^ Trimester | 0.50*** | -- |  |  |  |  |  |  |
| **3** PSS – 2^nd^ Trimester | 0.57*** | XX | -- |  |  |  |  |  |
| **4** PSS – 3^rd^ Trimester | 0.44** | 0.41** | 0.63*** | -- |  |  |  |  |
| **5** RADS – 2^nd^ Trimester | 0.37 | 0.45 | 0.12 | 0.04 | -- |  |  |  |
| **6** RADS – 3^rd^ Trimester | 0.43** | 0.36* | 0.27 | 0.27 | 0.63*** | -- |  |  |
| **7** Cortisol – 2^nd^ Trimester | 0.09 | 0.18 | 0.06 | 0.23 | -0.14 | 0.03 | -- |  |
| **8** Cortisol – 3^rd^ Trimester | -0.19 | -0.25 | -0.01 | -0.11 | 0.35 | -0.07 | 0.51* | -- |
| ^a^The value is expressed as ρ*,* where ρ is Pearson’s correlation  *p<.05; **p<.01; ***p<.001(Bonferroni corrected significance level) | | | | | | | | |
